# *Errare Humanum Est* (To Err Is Human): A Mentalizing Breakdown in the Therapeutic Work With an Adolescent

**DOI:** 10.3389/fpsyg.2021.789120

**Published:** 2021-12-09

**Authors:** Maria Wiwe

**Affiliations:** Karlstad University, Karlstad, Sweden

**Keywords:** intersubjectivity, mentalizing, repair, rupture, analytic impasse, complementarity

## Abstract

The therapeutic stance in therapies conceptualized by the two-person psychology (Wachtel, 2010) binds the therapist to genuine self-scrutiny. The concepts of transference and countertransference are viewed as jointly constructed endeavors between therapist and client, wherein the therapist needs to be aware of her contribution to difficulties arising in the therapeutic dyad. Different conceptualizations of this therapeutic technique have been eloquently described elsewhere throughout the years in terms of intersubjectivity (Stern, 2005; Aron, 2006), mentalizing (Fonagy and Bateman, 2006), mindfulness-in-action (Safran et al., 2001), rupture and repair (Newhill et al., 2003), and epistemic trust (Fonagy and Allison, 2014). These concepts will be presented interchangeably with a clinical vignette delineating a rupture in the therapeutic work with an adolescent. Finally, the article concludes with a discussion of identifying non-mentalizing modes (Allen et al., 2008) within the therapist to get back on track and restore epistemic trust (Fonagy et al., 2014) in the therapeutic relationship.

## Introduction

This article addresses the concepts of rupture and repair in a therapeutic process with an adolescent. Also, concepts connected to rupture and repair (Safran et al., 2001) like complementarity, analytic impasse, intersubjectivity and the third, eloquently described elsewhere by Aron (2006), will be discussed. The concept of intersubjectivity will be used interchangeably with mentalizing, always excellently described and defined in countless articles and books by the prominent fathers of the mentalizing theory themselves; Peter Fonagy and Anthony Bateman. The aim of this article is primarily to present reflections around mistakes on the part of the therapist. To work as a therapist is an assignment loaded with responsibility, sometimes making us fearful of doing or saying the wrong things or lacking in judgment. The fear itself might prevent us from learning from our mistakes and missing out on opportunities to deepen the working alliance with our clients.

To begin with, a short presentation of the meaning of intersubjectivity and its relatedness to mentalizing. Then an introduction of the young boy in therapy, followed by the rupture between him and his therapist, wherein the therapist fails to stay on the mentalizing course with the young person. After that, a clinical and theoretical discussion will follow concerning how to recognize own mentalizing failures, insecurity and pressure to reinstall intersubjectivity in the therapeutic work once it is lost. The article will end with concluding remarks on the importance of repairing ruptures in the therapeutic alliance.

### Intersubjectivity

In one of her articles from 2003, Karlen Lyons–Ruth contrasts the psychological two-person theories with theories that emphasize intrapsychic defense processes and argues that psychological defenses are a matter of intersubjectivity, born out of attachment relationships. According to Lyons-Ruth, our psychological defenses arise from the tension between the individual’s fear arousal and the response from the individual’s central attachment figures. Hence, coming into existence as a psychic being involves interplay with another human being, born out of intersubjectivity (Lyons-Ruth, 2003).

Intersubjectivity can be described, in Winnicott (1971) terms, as a transitional space between individuals allowing for the possibility of seeing the other as a subject in its own right with a meaningful inner life worthy of respect and genuine interest. Intersubjectivity is the opposite of relating to someone else as an object (Stern, 2005). The vital importance of recognizing the subjective experience of another is a core component of the mentalizing concept as well (Fonagy and Allison, 2014). The two concepts of intersubjectivity and mentalizing are highly related, even though mentalizing draws on extensive research from the fields of social cognition, attachment theory, evolutionary psychology and neurobiological research. The research on mentalizing is extensive and has vital importance for understanding human suffering and the importance of resilience (Sharp et al., 2009; Fonagy and Campbell, 2017; Choi-Kain et al., 2020). It is out of scope for this article to go into detail about this highly valued research.

### Mentalizing

Like the concept of intersubjectivity, mentalizing too is a profound social construct (Allen, 2018). Compared to mindfulness and empathy, two usually well-known concepts, mentalizing is always about the self in relation to others (Allen et al., 2008). When one is mindful, one tunes into one’s own unique experience; when being empathic one puts oneself into the shoes of another. When mentalizing, one tries to do both simultaneously to make sense of one’s emotional and relational world; hence mentalizing sits between the two (Allen, 2018).

Mentalizing is a unique human capacity, a simple yet complicated enterprise. Humans engage in mentalizing essentially without notice; it is automatic, implicit, and requires no effort. However, in times of emotional arousal, the mentalizing capacity decreases, and it demands our attention to stay on track and not lose sight of what is going on at an emotional and relational level. The opposite pole of the automatic one is then required; the explicit mentalizing using different brain areas is more reflective and persevering. The concept consists of another three polarities; affect-cognition, self-other, inner-outer; all parts of the polarities are underpinned by different neural pathways (Allen et al., 2008). Another crucial component of the mentalizing concept is the ability to couple and uncouple what is in one’s mind (fantasy) versus what is observable in reality, which is equivalent to imagining, with a low degree of certainty, the possibilities for others’ mental states and experiences (Choi-Kain et al., 2020). Briefly, a mentalizing brain is a brain in balance regarding these poles. Considering this, it is natural that we all fall prey to an imbalance in our mentalizing capacity from time to time.

Accordingly, therapists need to be sensitive to and recognize when we fail in our mentalizing ability since our clients need our balanced brains to help them make sense of their own emotional and social difficulties. They need us to be in tune with them, seeing the world from their perspective, taking a genuine interest in their emotional and social struggles. When therapists lose their mentalizing capacity, the readiness to own up to that assignment goes out the window.

### Effective and Ineffective Mentalizing

The mentalizing capacity varies from effective to ineffective (Choi-Kain and Gunderson, 2008) depending on the emotional arousal and level of pressure at hand. Still, in ineffective mentalizing, it is less arduous to get back to the effective mentalizing mode by pausing, being curious about what might be going on, and recognizing the ineffective mentalizing. Yet, once the individual has reached their tipping point, where the unique attachment history converges with the pressure level, they tip into non-mentalizing, and the way back to mentalizing becomes considerably more complex than from ineffective mentalizing to more effective mentalizing. The emotions leading up to the tipping point activate the individual’s attachment system, leading to a deactivation of the capacity to mentalize (Fonagy and Luyten, 2009). From this point, a re-emergence of non-mentalistic modes of representing subjectivity take place; psychic equivalence, teleological stance, and pretend mode (Allen et al., 2008).

Due to the deactivation of non-mentalizing, the individual becomes caught up in their own emotional world prevented from shifting perspective. The anxiety rises, and there is a loss of control. Attempting to recover control, we become too certain that our perspective takes precedence; we lose touch with the inherently uncertain reality. The higher the degree of certainty, the less flexible and realistic models of other people’s minds and the higher the risk of interpersonal difficulties (Fonagy and Target, 1996). According to the poles mentioned earlier, the non-mentalizing is always a sign that the brain has lost its balance, and we need to work our way back from prementalizing to mentalizing again.

### Prementalizing Modes

Psychic equivalence is one of the prementalizing modes, often referred to as non-mentalizing, which all characterize a mentalizing breakdown; the other two are called the teleological stance and pretend mode. Psychic equivalence is characterized by the absence of a barrier between the inner and the outer world. Emotions are experienced as reality. There are no other perspectives available than the personally felt one, whereas the teleological stance is action-oriented, focusing on change in the concrete outer world; emotions become actions instead of being processed and reflected upon (Allen et al., 2008). Pretend mode is the prementalizing mode, where there is no link between fantasy and reality (Luyten and Fonagy, 2015). There is no hierarchy between the three non-mentalizing modes, and one can end up in all three of them simultaneously. The brain gets out of balance and prevents the person concerned from making realistic models of mind, her own and others. Hence, therapists must be aware of their fluctuating mentalizing capability to be as helpful as possible for the client. Once the therapist is more ineffective in her mentalizing or has reached her tipping point, the risk is imminent that she will misread her client (Bateman and Fonagy, 2016).

Following this, a clinical case will be presented, in which the therapist falls prey to non-mentalizing and needs to work her way back to make realistic models of her and her patient’s mind. The case is a case of mine, although essential details have been changed why it is impossible to recognize the young person.

### Clinical Vignette

Noah, 18 years of age, presents clinically with borderline personality disorder features and vulnerable narcissistic traits. Struggling with affective instability, low impulse control and interpersonal difficulties, core features of the BPD diagnosis (Lieb et al., 2004), Noah turns to self-harm and acting out toward others in his immediate social context to regulate internal storms of emotions. Noah’s narcissistic traits demonstrate vulnerability concerning real or perceived setbacks entailing intense emotions of disappointment in himself and life in general. He is sensitive to competitive contexts and struggles to feel proud even when he is excelling.

His therapist often finds him in a dissociative state of mind due to raw, negatively socially loaded emotions like rejection, humiliation, disappointment and envy, triggered in interpersonal contexts. There is intensive therapeutic work to help Noah connect emotionally, and the treatment tends to be charged with solid countertransference feelings.

In terms of resilience, Noah is academically talented determinedly working hard in this area of his life. In social contexts where he feels secure and safe, his capacity for engaging in intersubjectivity is high. Still, his mental state can shift rapidly from this well-functioning reflective stance to more rigid ways of functioning, especially when experiencing the emotions mentioned above. Noah’s evident capacity to sometimes shift perspective and take a reflective stance on himself, his actions, and his impact on others bring about high expectations in the therapist, sometimes making her misjudge his mental state and underlying vulnerability.

Over a period, Noah and his therapist have worked consistently, aiming at improving his impulse control. Still, the last few weeks, the situation had been of a stalemate, Noah struggled in this area, and the therapist got more frustrated and worried he would end up harming himself or someone else. Before this particular session, the therapist received a call from Noah’s social secretary telling her that Noah, his girlfriend and his family had endured a rough morning ending in Noah smashing a window with his bare hand. The social secretary asked the therapist if she worked with Noah’s low impulse control, as was agreed upon in his client care management.

Entering the session, Noah (in a non-mentalizing state of mind) is anxiety-ridden, looking down on the floor, placing himself immediately in the chair intended for him, not taking his jacket off as he usually does. His hand is in bandages.

Noah mumbles; “I smashed another window.”

Therapist (feels pressured, ineffective mentalizing); “Yeah, I heard, I got a call from social services.”

Noah (teleological stance/psychic equivalence); “Huh, I bet they wanna throw me out of treatment, everyone being fed up with me.”

The therapist responds to Noah’s non-mentalizing mode by tipping into non-mentalizing as well and displays a grave facial expression addressing the bandaged hand (psychic equivalent/teleological); “You are hurt.”

Noah frowns, looks out of the window, replacing himself in the chair (psychic equivalent/pretend mode); “I’m fine, I’m good actually. It is just a few scratches.”

Therapist (pretend mode); “That’s a relief, that is good.”

Noah (pretend mode); “Yeah, I don’t want any more scars.”

Therapist (with a bit of an edge in her tone of voice, pretend mode/psychic equivalent): “Ah, I see. Yeah, I don’t think it was a good idea, smashing your hand through the window.”

In response to this comment from the therapist, Noah distinctly shuts down, breaking eye contact; he loses his posture and leans forward, hiding his face behind a curtain of hair.

The therapist responds to Noah shutting down with becoming fearful and desperately wants to get him out of his enclosed state of mind. Therefore, she leans toward him to “reach him” (teleological; “he must know I care!”), only with the result of further overheating Noah’s attachment system. Hence, Noah withdraws even more (pretend mode).

Therapist leaning forward; “Noah?” Noah is hiding, his hair covering his face, rocking himself back and forth (psychic equivalent/pretend mode): “You said it wasn’t a good idea.”

Therapist (still absorbed by fear and her perspective on what have unfolded, not yet capable of being genuinely interested in Noah’s perspective); “Well, I don’t think it was a good idea because I don’t want you to get hurt.”

Noah (teleological): “Huh, you only care about the broken window, and I already know that I suck; I don’t need you to tell me that.”

Therapist (still fearful and unable to identify what is transpiring between them, therefore demands him to listen to her, teleological/psychic equivalent); “No, no, listen to me; I said that because I don’t want you to harm yourself.”

Noah (psychic equivalent); “Whatever, I’m such a loser; everything I do is crap.”

Therapist (teleological/psychic equivalent); “No, Noah, listen to me, you have so many great ideas, but this one, smashing the window, was not my favorite idea; that is just it.”

Noah (psychic equivalent); “I suck.”

The therapist leans forward even closer, trying to catch Noah’s eyes, which only heightens his arousal. Noah is pressing himself closer into the chair, hiding even more behind his curtain of hair. Suddenly, when he also starts to shake his legs intensely, the penny drops for the therapist. She becomes aware of her persecutory act on Noah, and she starts to pay attention to her mental state, acknowledging her anxious emotions and non-mentalizing stance.

Therapist (another tone of voice; softer and calmer); “Noah, gosh, I’m sorry, you know I was too hard on you (taking responsibility). I can see I had kind of an edgy voice telling you about me thinking it was a bad idea smashing your hand through the window. I’m sorry.”

Noah stops sobbing.

Therapist (sounds genuinely contrite); “I’m sorry I didn’t get how much you struggled and how brave you were telling me what happened. You know what, I guess I sometimes get stuck in the view of you as a young person functioning very well, and I lose sight that you are also struggling hard from time to time. When I get stuck like that, I get frustrated and even a bit irritated. Do you see what I mean?”

Noah nods.

Therapist; “I guess I become really demanding then, huh?”

Noah listens.

Therapist (playing with a dark, demanding voice, shaking her index finger in the air); “Like this Noah, this is me, isn’t it; Young man, I know you can do much better; you better don’t let me down.”

Noah smiles.

Therapist; “You know, if I were in your shoes, hearing that edgy voice, I would have been, you know, kind of angry, like ‘what does she know about anything?’. But that is me; it doesn’t necessarily need to mean that you got angry?”

Noah shrugs.

Therapist; “You know, like ’who the heck does she think she is?!”

Noah giggles.

Therapist; “Still, I wonder, did you, did you feel angry with me?”

Noah shrugs.

Therapist; “You see, I am wondering if you are and if you are actually protecting me from your anger hiding behind the curtain of your hair?”

Noah looks up.

Therapist; “You see, I think you are the brave one here, the one who really struggles, trying to protect me from your anger, is that so?”

From here, the therapeutic dialogue took a turn for the better since the therapist finally identified her non-mentalizing spin due to non-recognized emotions. Taking the pause, slow things down, owning up to her mistake and finally tuning into Noah’s experience at the moment, Noah felt recognized and seen, and they could start over.

From now, a discussion of the breakdown of intersubjectivity, non-mentalizing therapist, and how to break the vicious non-mentalizing cycle.

## Discussion

The following section primarily discusses the mentalizing failure of the therapist and the mental process in the therapist to help her get back on the mentalizing track. There will be some comments on Noah’s mentalizing breakdown as a consequence of the earlier turmoil in his social life and in response to the non-mentalizing attitude of hos therapist.

### Be Aware of Pressure

Mentalizing starts with paying attention to mental shifts; following that comes the awareness of our mental states’ impact on other people. Noah’s therapist, who just hung up the phone after the call from the social secretary, is not paying attention to her shift in mode. Instead, she is unaware of the pressure she is under, stuck in the automatic, affective dominated mentalizing where she is ineffective in her mentalizing capacity. She responds to the pressure from the social network around Noah, wanting her to do something, by becoming frustrated and solution-focused – she is not pausing, checking her mentalizing, asking herself if she is capable of empathizing with Noah’s emotional position in order to help him generate alternative perspectives on the situation eventually. Thus, she starts by viewing Noah through a lens of frustration, pressure and disappointment even, why she misses out on picking up on clues about Noah’s anxious mode and his avoidant gaze.

### Reaching the Tipping Point

Further, apart from responding to the stress conveyed by the network, the therapist is also responding to Noah’s psychic equivalent mode. Noah is consumed by anxiety, shame, and guilty feelings prevented from cognitive reappraisal of the situation. As is familiar with adolescents, he becomes his emotions – feeling bad is being bad (McRae et al., 2012). Noah’s state of mind is influencing the therapist, and these emotions become her reality as well; she reaches her tipping point and enters the realm of psychic equivalence where there is no room for alternative perspectives (“I’m a bad therapist, this is too much, I’ve worked so hard with Noah’s impulse control, why isn’t he listening to me? This happens every time,” “I should look for a career change”). This dark ruminating quality of the mental process is a signal of non-mentalizing. When recognizing these mental states, the note to self is that the brain needs to recover its balance. Unfortunately, it is not always enough to take a deep breath or count to ten since there is a need for a biological switch in the brain to see the interaction from a reality-based point of view. The biological switch can be brought about by reminding oneself as a therapist to continually check in on one’s mentalizing level before and during sessions. Then the identification of ineffective or non-mentalizing modes will be easier to detect. The switch often requires a good pause, for example, speaking out loud, “I can’t really think straight, I guess you notice? Let me take a pause so I can restart my brain. Do you think I come across as different today? I think I need your help to put myself right again, is that okay with you?” In taking the pause and slowing down the pace, inviting the client to share the mental work of the therapist as a model, the therapist aims at preventing a stalemate in the therapeutic process.

However, let us stay with Noah and his therapist in their struggle for a while. Here, the therapeutic dyad is stuck in a deadlock, a therapeutic impasse entailing the therapist’s inability to see Noah from the inside out.

### The Therapeutic Dyad in a Deadlock

The crux with non-mentalizing is that it will only worsen if it does not come to a halt. Non-mentalizing begets non-mentalizing, just as mentalizing begets mentalizing (Allen et al., 2008). The therapeutic dyad is in a deadlock, a vicious cycle of non-mentalizing, which the therapist is assigned to unlock. She reacts to Noah’s psychic equivalence by becoming more frustrated and captivated by hopelessness, and they share the same predicament characterized by raw unforgiving emotions. Thus, the subsequent mental shift from psychic equivalence into the pretend mode is logical. The prementalizing mode of psychic equivalence often entails an unintentional switch into pretend mode where emotions are more out of reach; in the pretend mode, there seems to be no emotional contact either with oneself or with someone else. Pretend mode produces mental states characterized by emptiness and meaninglessness and tends to take a form of intellectualization or psycho-babbling (Allen et al., 2008), where there is no therapeutic gain; instead, the therapeutic process is equivalent to a wheel spinning in the sand (Fonagy and Target, 2000).

### Spinning in the Pretend Mode

Not paying attention to the impasse, the tip into non-mentalizing, stops the therapist from making it explicit, making meaning out of it, and exploring the potential effects it might have had on Noah; there is no reparation (Newhill et al., 2003). Instead, she gets caught up in her distressing emotions and starts to operate in pretend mode. Her stress is augmenting, and she starts to feel fearful.

The indication of the shift into the pretend mode is when they start to talk about Noah and his hand. The dialogue between them is then distinctly avoidant of emotions; they are both refraining the emotional content in Noah’s statement about him smashing the window. Pretend mode is commonly characterized by bypassing the elephant in the room, such as the probability that it might be more to this than feeling good when one recently smashed a hand through a window due to a fight with loved ones.

Eventually, the emotions arise again, and the non-reflective therapist tries on a superficial level to say something nice, although her unawareness and heated state of mind comes across through her edgy voice. Not being attentive enough to the emotions running high and her pertinent ineffective mentalizing interfere with her ability to notice her edgy voice. Hence, she is not able, yet, to fully grasp its impact on Noah.

Still, being caught up in her distress clouding her view, she perceives nothing but her own emotions. Again, she is not paying attention to her stern facial expression, which puts Noah in trouble. Boys tend to perceive severe faces as anger directed toward them (Tahmasebi et al., 2012), causing an intensifying of Noah’s non-mentalizing, perceiving himself as judged and not wanted, his self-hatred making him identified with his alien self (Rossouw and Fonagy, 2012; Rossouw, 2015), and he shuts down.

Here we have a fearful therapist, Noah, still stuck in psychic equivalence where self-hatred fragments his mind, the therapist trying to persuade Noah she only meant well, not yet aware she is primarily working in her favor (pretend mode), trying to decrease her anxiety.

Let us leave the therapeutic dyad of Noah and his therapist and turn to the invaluable contributions from different prominent clinicians and researchers whom all have contributed immensely to the everyday life of ordinary clinicians trying to make the therapeutic climate for patients (and themselves) alive, flourishing and productive.

### Theoretical Frame

The frontal figures of the mentalization-based theory, Bateman and Fonagy (2006), describe the importance of the mentalizing stance on the therapist’s part during clients’ affective storms. When something goes wrong in the therapeutic interaction, the intersubjectivity and the mentalizing capacity are compromised concerning both client and therapist. Misunderstandings and non-matched communication threaten to intensify emotional arousal, entailing an overheated attachment system. It then becomes impossible for the client to mentalize his state of mind and the mind of the therapist. This situation prevents the client from identifying and expressing emotions and needs and seeing the situation from different angles. The mind ends up being a place without meaning, only full of pain, leaving the client in absolute need of the therapist’s balanced mind to recuperate their own. That is why Bateman and Fonagy describe the importance of the therapist taking responsibility for the interactive negativity that has unfolded (Bateman and Fonagy, 2006), aiming at helping the client to make the mental world meaningful again. They emphasize the importance of the mentalizing stance on the therapist’s part, especially during clients’ affective storms. The mentalization-based treatments frame the therapeutic dyad as a variant of an attachment relationship. Working with insecurely attached clients, the risk for overheating the attachment system due to negative communication arises. Sensitivity in the attachment system can easily and rapidly cause an overheating of the attachment system within the client, paving the way for affective storms and loss of reflective capacity (Fonagy and Bateman, 2008). So, what can we as therapists do then, when we too have lost access to our balanced minds?

### Finding the Third

In the beautifully written article “Analytic impasse and the third - Clinical implications of intersubjectivity theory” (2006), Lewis Aron described the recognizable situation of feeling stuck in the therapeutic situation, not getting anywhere or knowing what to do to get out of it, frustrated emotions augmenting. Aron wrote about this deadlock as an analytic impasse constituted by complementarity in human dyads. To illustrate this situation, Aron used the metaphor of a compass needle fixed either on North-South or East-West. The rapid and flexible movements of the needle adjusted to its surrounding context are replaced by only a straight stagnant line. Any movement to another cardinal point is conspicuous by its absence. According to Aron, this is a frequent predicament in ongoing therapies, which is sometimes why therapies interrupt prematurely (Aron, 2006).

Jessica Benjamin is another relational theorist and clinician explaining the concept of complementarity (2004). Instead of the compass needle, she introduces the seesaw as a metaphor for the analytic impasse. Accordingly, Aron and Benjamin refer to complementarity as causing an analytic impasse, linearity without breathing space. There is no psychic air in the impasse, no room for reflection or possibility for the dyad members to relate to each other in an intersubjective or mentalizing way. By the elegant metaphor of the seesaw, Benjamin illustrates the human dilemma of complementarity, appearing regularly in therapeutic processes. The illustration of the seesaw captures how human dyads tend to get stuck on each end quickly. Once stuck, the dyad enters the doer-done relationship (Benjamin, 2004). In the doer-done relationship, also called the push-pull exchange, one part of the dyad does to the other, and the other is being done to Benjamin (2004). One is playing the role of perpetrator, the other one the victim. One is playing the part as passive, the other one as active, one is female, the other male, one is positive, the other is pessimistic, and so forth. Benjamin (2004) and Aron (2006) suggest that if there is a movement in this predicament, it is only about switching roles.

Noah and his therapist are stuck on the seesaw (Benjamin, 2006). The complementary dilemma they are in forces one to take on the role as a perpetrator “you ought to listen to me, I did nothing wrong, I was only caring about you,” the other the victim “you are just like everyone else, you don’t care about me.” Then they switch roles, the perpetrator becoming the victim “hey, I am trying everything here, why are you not listening to me, huh? I’m the good guy!” and the victim becomes the perpetrator “I am not looking at you, I am not talking to you, I am most definitely not going to listen to you.” Aron (2006) suggests, on the seesaw, in complementarity, there is no space for psychic improvement; the dyad will suffocate unless something happens.

In a deadlock between therapist and client, the client and the therapist are hindered by their blind spots (Bonovitz, 2009). They are both communicating through their historically relational filter (“Why isn’t he listening to me?,” “Why is everyone on me?”) entailing the imminent risk of starting to objectify (“this is so typical adolescents,” “adults don’t get it”) one another (Bonovitz, 2009). Several relational thinkers (Ogden, 1999; Allen, 2006; Aron, 2006; Wachtel, 2007) encourage therapists to be alert to the danger of objectifying the client.

Benjamin Wolstein, who coined the notion of the interlock between therapist and client in 1959, is reviewed by Bonovitz (2009). According to Bonovitz (2009) Wolstein suggests that objectifying leads to both parties being locked in their countertransferentially interpretations of the other, which primarily creates a reactive unit in which neither can move in a new direction. They are stuck in relation to each other where all psychological development ceases to exist. Simultaneously, this impasse is taking the character of a reciprocal endeavor between the two parties since the therapeutic impasse aims at keeping the anxiety at a low level (Bonovitz, 2009). Moving out of the reactive unit creates anxiety, as change tends to do; hence, therapist and client are protecting each other from their anxiety by not moving out of the reactive unit. Still, the impasse needs to be abandoned, especially if a premature ending of therapy can be prevented. Something needs to happen; the reactive unit needs a movement that pulls them out of the impasse.

Furthermore, Wolstein (Bonovitz, 2009) means that this movement stirring up within the impasse requires a more intense level of relating between the two (Bonovitz, 2009). A relating characterized by addressing what is going on in the here-and-now between therapist and client. The therapeutic focus needs to be on the emotional reaction to each other in their immediate presence if the reactive unit will move away from viewing one another only through the lens of historical projections. Wolstein suggests that both need to change (Bonovitz, 2009). The therapist changing is the core component in the therapeutic intervention. Even the therapist needs to discover new sides of themselves viewed through the eyes of the client. Wolstein encourages the therapist to face up to their narcissism and bear the discovery of new unexplored sides of themselves and own up to their limitations (Bonovitz, 2009). The responsibility is on the therapist to take the first step toward discovery and let themselves be more vulnerable (Aron, 2006).

### The Creation of the Third

Benjamin (2006) resonates in line with Wolstein that therapists and clients cause ruptures and impasses in the therapeutic dyad and are both initiating repair. Both parties withdraw and reconnect. Benjamin defines this process as a dyadic dance, where the risk of impasses and complementarity is a constant threat to a dynamic and vivid therapeutic process.

The doer-done relationship mentioned above needs to be observed and recognized to create a thirdness within the dyad. The thirdness being a position within the dyad consisting of mentalizing the relationship (Bateman and Fonagy, 2016). As Wolstein, Benjamin (2006) writes about the pain in creating the third and the inevitable and necessity of this pain being born in the third space. It is the anxiety that Wolstein is speaking about when the dyad becomes triadic *via* metacommunication about the relationship, i.e., mentalizing the relationship.

Furthermore, Benjamin points to the dilemma of how easily a therapist risks getting into shared dissociation with the client. Shared affectivity (Jurist, 2005) of, for example, emotions of profound abandonment and meaninglessness. She claims that if the third is not introduced within the dyad, the consequence might be that therapist and client are sharing a painful historical experience from the client’s life. An experience then prevented from being verbalized, made conscious and fully experience, why it will stay operating at an unconscious level. Benjamin calls the unprocessed non-verbal emotionally historical experience the death in the meaningless abandonment (Benjamin, 2006). She underlines the importance of the therapist’s capacity to step outside the historical drama, which is crucial to capture a realistic view of the impasse. Stepping out of the dyad wondering “what is happening?” creates the third, and one stops playing a destructive role in the relived drama.

Additionally, Benjamin (2006) conveys that the solution to the impasse is the creation of the third *via* metacommunication. It requires that the therapist move out of the impasse; it is impossible to stay within the dyadic interaction. The step to the side is vital in order to create the third.

Finally, Benjamin (2006) clarifies that it is not enough that the third is born only in the therapist’s mind; she means that it is required to happen in joint attention (Tomasello, 1995; Fonagy et al., 2007) with the client. The step to the side is driven by the therapist being vigilant to her transference, conveying it to the client to break out from complementarity (Benjamin, 2006).

### Breaking the Complementarity

In the presented case example, the therapist initiates the third’s creation when she eventually finds herself in her persecutory act toward Noah. Noah’s evident bodily demonstration (shaking his legs) of his predicament finally gets to her. She then shifts from being stuck in automatic mentalizing and starts to observe the situation more from the outside. Her emotional reactions get to her; she becomes aware of her pressured, non-mentalizing state, feeling a shiver of shame, realizing the edge in her voice was born out of frustration and pressure.

By taking a step back mentally and physically, she starts to wonder if her frustration causes the behavior he is now displaying. Furthermore, in the here and now, she experiences him behaving difficultly and that he is hard to reach, and she now thinks that it might be a way for him to protect her from his anger. Is he struggling to preserve their relationship when she is the one who risks it? Thus, in the sense of joint attention (Tomasello and Farrar, 1986), she decides to invite him to share her thoughts; thereby, she initiates the reflective metacommunication on what might have been transpiring between the two on an emotional level. She starts to push them of Benjamin’s seesaw.

## Conclusive Remarks

In the reparative work, the therapeutic focus needs to be on the emotional reaction to each other in their immediate presence if the reactive unit will move away from viewing one another only through the lens of historical projections. Wolstein suggests that both need to change (Bonovitz, 2009). The therapist changing is the core component in the therapeutic intervention. Even the therapist needs to discover new sides of herself viewed through the eyes of the client. Wolstein encourages the therapist to face up to their narcissism and bear the discovery of new unexplored sides of themselves owing up to their limitations (Bonovitz, 2009). The responsibility is on the therapist to take the first step toward discovery and let themselves be more vulnerable (Aron, 2006).

*Via* mentalizing the relationship, the therapist starts to crate the third, taking responsibility for the breakdown of intersubjectivity. The aim is not to be spot on in uncovering the relational pattern of the shared dissociation. The aim is to convey one’s awareness of one’s impact on the client and own up to that so that trust can be rebuilt in the dyad (Bateman and Fonagy, 2013).

In the same sense as Benjamin, Aron claimed that the dyad needs to evolve into a psychic triad. The interlock of the dyad restricts everyone’s behavioral repertoire in the interpersonal situation. If we do not manage to take a step to the side, we are all destined to one reaction; client and therapist, the stakes are high in this restricted repertoire of behavior. It should always be the therapist going; first, the responsibility is on the therapist, even though sometimes the client is the one initiating the repair. When they do, they shall be praised (Fonagy et al., 2019).

Benjamin is in good company (Bateman and Fonagy, 2004; Aron, 2006; Wachtel, 2007; Goodman et al., 2009; Eubanks et al., 2018), suggesting that the analytic impasses harbor an enormous potential for improving the therapeutic process. In explicit terms, Benjamin conveys that therapists should not do anything forbidden to cause or unlock the impasse. However, for some theoretical schools, they might need to do something forbidden to unlock the impasse; the use of self, the self-disclosure, the metacommunication, the reflection upon the hear-and-now, the mentalizing of the moment, and the relationship (Bateman and Fonagy, 2016) in order to get back to the intersubjective space. The dyad needs to transform into a triad, where the third party is the mentalizing of the moment and the relationship (Bateman and Fonagy, 2016), the reflection upon the experience we share at the moment. That seems to be the place where we build trustworthy relationships, not in flawless communication but the reparation of our human flaws.

## Author’s Note

MW is a Clinical Psychologist and Psychotherapist and qualified MBT-A therapist, supervisor and trainer as well as a certified MBT-F practitioner. Together with Trudie Rossouw, Maria is training and assessing new MBT-A supervisors at the Anna Freud National Centre for Children and Families. She is an experienced lecturer and supervisor in MBT-A and has written several books on the subject in Swedish.

## Author Contributions

The author confirms being the sole contributor of this work and has approved it for publication.

## Conflict of Interest

The author declares that the research was conducted in the absence of any commercial or financial relationships that could be construed as a potential conflict of interest.

## Publisher’s Note

All claims expressed in this article are solely those of the authors and do not necessarily represent those of their affiliated organizations, or those of the publisher, the editors and the reviewers. Any product that may be evaluated in this article, or claim that may be made by its manufacturer, is not guaranteed or endorsed by the publisher.
